# Relationship between Psychological Stress Determined by Voice Analysis and Periodontal Status: A Cohort Study

**DOI:** 10.3390/ijerph19159489

**Published:** 2022-08-02

**Authors:** Takayuki Maruyama, Daisuke Ekuni, Masakazu Higuchi, Eiji Takayama, Shinichi Tokuno, Manabu Morita

**Affiliations:** 1 Department of Preventive Dentistry, Okayama University Academic Field of Medicine, Dentistry and Pharmaceutical Sciences, Okayama 700-8558, Japan; dekuni7@md.okayama-u.ac.jp (D.E.); mmorita@md.okayama-u.ac.jp (M.M.); 2 Advanced Research Center for Oral and Craniofacial Sciences, Okayama University Dental School, Okayama 700-8558, Japan; 3 Department of Bioengineering, Graduate School of Engineering, The University of Tokyo, Tokyo 113-8655, Japan; higuchi@bioeng.t.u-tokyo.ac.jp (M.H.); or s.tokuno-wm2@kuhs.ac.jp (S.T.); 4 Department of Oral Biochemistry, Asahi University School of Dentistry, Gifu 501-0296, Japan; takayama@dent.asahi-u.ac.jp; 5 Graduate School of Health Innovation, Kanagawa University of Human Services, Kanagawa 210-0821, Japan

**Keywords:** periodontitis, psychological stress, voice analysis, prospective cohort study

## Abstract

In modern society, evaluation and management of psychological stress may be important for the prevention of periodontal disease. The purpose of this study was to examine the relationship between psychological stress (vitality and mental activity) evaluated by Mind Monitoring System (MIMOSYS) and periodontal status. Forty students of Okayama University underwent the oral examination and self-reported questionnaire on the first day (baseline) and the 14th day (follow-up). Voice recording was performed every day with the MIMOSYS app during the whole study period. The participants completed the Patient Health Questionnaire (PHQ)-9 and Beck Depression Inventory (BDI) at baseline and at follow-up. Spearman’s rank correlation coefficient was used to determine the significance of correlations among variables. The PHQ-9 and BDI scores were negatively correlated with vitality in the morning. Change in vitality in the morning was significantly correlated with changes in periodontal inflammation. Mental activity was significantly correlated with change in mean probing pocket depth. This result shows that measurement of psychological stress using a voice-based tool to assess mental health may contribute to the early detection of periodontal disease.

## 1. Introduction

Periodontal disease is a chronic inflammatory disease caused by oral bacteria [1]. Since periodontal disease is one of the major causes of tooth loss [2], prevention, early detection, and early treatment of periodontal disease are important.

A relationship between psychological stress and periodontal disease has been reported. Anxiety and depression are associated with altered immune responses and may increase susceptibility to periodontal disease [3,4,5,6]. In modern society, evaluation and management of psychological stress may be important for the prevention of periodontal disease.

In the previous studies, objective methods using biomarkers and subjective methods using questionnaires were used to evaluate psychological stress. Measurement of cytokines in saliva has been shown to be useful as an objective method of assessing psychological stress [7]. The stress response is also associated with increased neural activity in the hypothalamus-pituitary-adrenal axis or the sympathetic-adrenal medulla axis, resulting in the release of cortisol from the adrenal cortex [8,9]. Therefore, it has been reported that the measurement of cortisol in saliva is useful as an evaluation of psychological stress [10]. In addition, salivary cortisol concentration is associated with periodontal pocket depth [11]. However, these methods are invasive, laborious, and costly because they require special measuring instruments or reagents. On the other hand, self-reported psychological questionnaires such as the Patient Health Questionnaire (PHQ)-9 [12] and the Beck Depression Inventory (BDI) [13] are generally used. Although these questionnaires are non-invasive and simple, the effects of reporting bias whereby respondents consciously underestimate or overestimate certain information, cannot be avoided. In the previous studies, depression diagnosed by the PHQ-9 was associated with periodontitis in one report [14], while in another report, it was not associated [15]. Furthermore, in one report, depression diagnosed by the BDI was associated with periodontitis [16], whereas in another report, it was not associated [17].

In contrast, it is empirically and academically known that changes in mood appear in facial expressions and voices [18,19], and studies have been conducted to evaluate depression and stress states using them [20,21,22,23]. In particular, voice analysis is non-invasive, does not require any special equipment, and can be performed easily and remotely with only smartphones. Furthermore, it has the potential to resolve reporting biases during self-administered psychological tests and can detect various psychiatric disorders. Therefore, voice analysis has been attracting attention in recent years. The authors have developed an index called “vitality” [24], which estimates the degree of mental health from the voice and have distributed it as a software development kit and a smart phone/web application (Mind Monitoring System: MIMOSYS, PST Inc., Yokohama, Japan) [25]. It is widely available to many companies and general users [26]. It is expected that this method can be used to monitor mental health daily and to avoid mental disorders such as depression.

We postulated that there is a relationship between psychological stress evaluated by MIMOSYS and periodontal status. Therefore, the purpose of the present study was to examine the relationship between psychological stress evaluated by MIMOSYS and periodontal status. The concept of the present study was to enable periodontally healthy people to detect psychological stress with MIMOSYS application and detect the exacerbation of periodontal status at early stage.

## 2. Materials and Methods

### 2.1. Ethics Statement

The protocol of this prospective cohort study was approved by Okayama University Graduate School of Medicine, Dentistry and Pharmaceutical Sciences and Okayama University Hospital, Ethics Committee (No. 1809-032). All methods were performed in accordance with the Declaration of Helsinki. Written, informed consent was obtained from each participant. Study reporting conforms to the STROBE guidelines.

### 2.2. Participants

Forty students of Okayama University participated in this study. Inclusion criteria were that participants must be over 20 years of age. Exclusion criteria were as follows: (1) participants with a medical history of systemic disease; (2) participants who had not received periodontal treatment within 3 months; (3) participants with a medical history of taking antibacterial, anti-inflammatory, or anti-allergic drugs within the past 2 weeks; and (4) participants with a history of smoking [27]. Each participant underwent the oral examination and self-reported questionnaire on the first day (baseline) and the 14th day (follow-up). Voice recording was performed every day with the MIMOSYS app during the whole study period.

### 2.3. Oral Examination

One dentist (D.E.) examined oral status at baseline and follow-up. Probing pocket depth (PPD) was measured at six sites (mesio-buccal, mid-buccal, disto-buccal, mesio-lingual, mid-lingual, and disto-lingual) on all teeth except third molars using a color-coded periodontal probe (CP-11 Color-Coded Probe, Hu-Friedy, Chicago, IL, USA). The percentage of sites with bleeding on probing (%BOP) was calculated. The periodontal inflamed surface area (PISA) was calculated using the Excel spreadsheet program [28]. Oral hygiene status (plaque control record, PCR) was measured after staining by erythrosine and recorded at four sites (mesial, distal, buccal, and lingual) in the cervical region of each tooth [29]. PPD was recorded and repeated within a two-week interval in two volunteers. Intra-examiner reliability of PPD evaluated by the kappa statistic was >0.8.

### 2.4. Self-Reported Psychological Questionnaires

The participants completed the PHQ-9 and BDI at baseline and at follow-up. The PHQ-9 is a self-reported questionnaire for assessing the severity of depression. The nine items on the scale are based on the nine diagnostic criteria for major depressive disorder. Each item is rated on a four-point scale from 0 (Not at all) to 3 (Nearly every day), and a higher total PHQ-9 score is associated with more severe depression [12]. The BDI is also a self-reported questionnaire for assessing the severity of depression. The BDI scale consists of 21 items, each item is rated on a four-point scale from 0 to 3, and a higher total BDI score is associated with more severe depression [13].

### 2.5. Evaluation of Voice-Based Mental Health

The participants received a smartphone with the MIMOSYS app downloaded and recorded fixed phrases three times a day (morning, afternoon, night) for 14 days using this app. There are 13 fixed phrases, which consist of phrases selected from the viewpoint of Japanese phonology, common Japanese phrases, and phrases that are thought to induce positive or negative emotions. The phrases that are thought to induce emotions were selected from those psychiatrists that were asked in their medical treatment. The voice analysis method used by MIMOSYS is based on sensibility technology, which analyzes patterns of changes in the fundamental frequency of voice data to evaluate the degree of calmness, anger, joy, sorrow, and excitement [26]. Then, MIMOSYS uses the sensibility technology data to calculate “vitality”, which quantifies mental health, and “mental activity”, which indicates fluctuations in vitality for two weeks. Vitality and mental activity were calculated using the algorithm as values from 0 to 1, and extremely low or high values have been determined to represent mental health abnormalities.

### 2.6. Bias

To minimize measurement bias, one consistent dentist examined oral status, and calibrated instruments were used.

### 2.7. Sample Size Estimation

Sample size estimation was not performed because there were no previous studies that investigated the relationship between psychological stress evaluated by MIMOSYS and periodontal status. Therefore, this was a pilot study.

### 2.8. Statistical Analysis

Changes in each parameter over 14 days were calculated by subtracting the baseline value from the follow-up value. Spearman’s rank correlation coefficient was used to determine the significance of correlations among variables. All analyses were performed using SPSS 25.0J for Windows (IBM Japan, Tokyo, Japan). Values of *p* < 0.05 were considered significant.

## 3. Results

**Figure 1:**
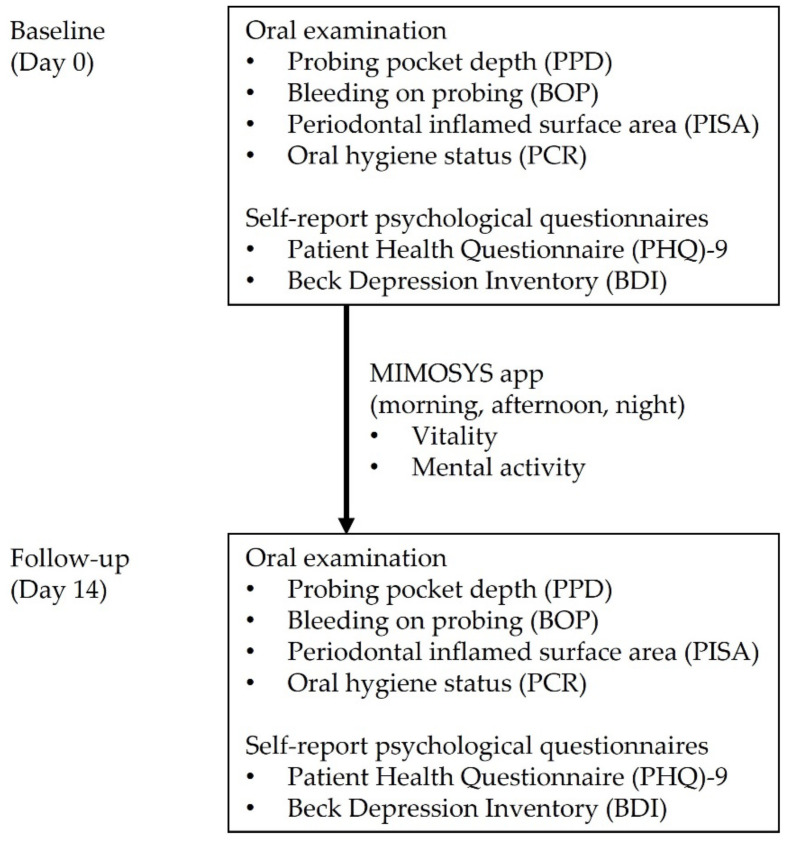
The protocol of this study.

Figure 1 shows the protocol of this study. No participants dropped out during the study period. No participants changed their oral hygiene behaviors, such as frequency of tooth brushing every day or using interdental brushes.

The results of oral examination, the self-reported psychological questionnaire, and vitality and mental activity at baseline and follow-up are shown in Table 1.

The correlations between the self-reported psychological questionnaire score and vitality or mental activity are shown in Table 2. The PHQ-9 and BDI scores were negatively correlated with vitality in the morning (ρ = −0.221, *p* = 0.025; ρ = −0.219, *p* = 0.025, respectively).

**Figure 2:**
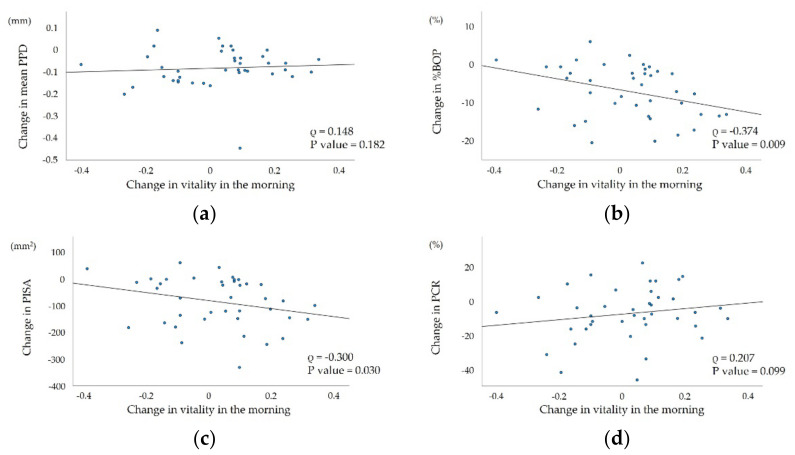
Correlation between change in vitality in the morning and (**a**) change in mean PPD; (**b**) change in %BOP; (**c**) change in PISA; and (**d**) change in PCR.

The correlations between mental activity, changes in vitality in the morning, PHQ-9 and BDI scores, mean PPD, %BOP, PISA, and PCR are shown in Table 3. Change in vitality in the morning were significantly correlated with changes in %BOP and PISA (ρ = −0.374, *p* = 0.009; ρ = −0.300, *p* = 0.030, respectively) (Figure 2). In addition, mental activity was significantly correlated with change in mean PPD (ρ = −0.265, *p* = 0.049).

## 4. Discussion

The relationship between psychological stress evaluated by MIMOSYS and periodontal status was investigated, and it was found that vitality in the morning was negatively correlated with the scores of self-reported psychological questionnaires (PHQ-9 and BDI). Furthermore, change in vitality in the morning over 14 days was significantly correlated with the change in the degree of periodontal inflammation. To the best of our knowledge, this is the first study to show the relationship between psychological stress and periodontal status using a system for monitoring mental health using voice data.

In this study, vitality in the morning was negatively correlated with the scores of the PHQ-9 and the BDI. On the other hand, vitality in the afternoon, evening, and mental activity were not correlated with the scores of the PHQ-9 and the BDI. In a previous study, vitality determined using voice recordings was negatively correlated with the BDI score [30]. There are diurnal fluctuations in vitality, which are generally low in the morning and high in the evening [31]. In this study, the measurement time of vitality in the morning was close to waking up, with little variation in the measurement time. However, there was a great deal of variation in the measurement time of vitality in the afternoon or evening because every participant had different life events during the day. Among the evaluations of voice-based mental health using MIMOSYS, vitality in the morning is considered to be useful as an index for evaluating psychological status.

In the present study, change in vitality in the morning over 14 days was negatively correlated with changes in %BOP and PISA. On the other hand, changes in the scores of the PHQ-9 and the BDI were not correlated with changes in %BOP and PISA. The PHQ-9 and the BDI are self-reported psychological questionnaires; therefore, there may be reporting bias, in which respondents consciously underestimate or overestimate certain information. In contrast, vitality analysis monitors the acoustic features of the voice, and it estimates the involuntary effect of autonomic nerves on vocal cord movement and indicates mental status [25]. Therefore, reporting bias may be avoided. Changes of vitality in the morning are considered to be a useful indicator of changes in the degree of periodontal inflammation.

In the present study, mental activity was negatively correlated with the change in mean PPD. Mental activity indicates fluctuations in vitality over the last two weeks [26]. Periodontal disease is a chronic inflammatory disease that forms deep periodontal pockets over a long period of time. Furthermore, periodontal disease is a silent disease that, in the early stage, has fewer subjective symptoms and effects on quality of life than other oral diseases [32]. The present study showed that changes in mean PPD over a short period of two weeks were associated with mental activity. Measurement of mental activity may contribute to the early detection of periodontal disease.

There are some limitations related to this study. First, a detailed survey of lifestyle-related factors was not conducted in this study. In addition, although none of the subjects had bad oral habits or temporomandibular disorders, we have not considered the effects of bruxism on periodontal tissue. Therefore, confounding factors related to psychological stress may exist. Second, since this study was a short-term study lasting only two weeks, the results of long-term observations may differ in the relationship between periodontal status and psychological stress. Third, since this study was a pilot study with a small number of 40 cases, the correlation coefficients may have weakened. These correlation coefficients may be stronger in larger studies. Finally, the participants of this study were young and almost all had good periodontal health. Thus, one must be careful about generalizing the present findings.

## 5. Conclusions

Vitality in the morning was negatively correlated with the score of self-reported psychological questionnaires. In addition, change in psychological stress over 14 days was significantly correlated with changes in the PPD and the degree of periodontal inflammation. This result shows that measurement of psychological stress using a voice-based tool to assess mental health may contribute to the early detection of periodontal disease.

## Figures and Tables

**Table 1:** Characteristics of participants.

Variables	Baseline	Follow-Up
Mean PPD (mm)	1.91 ± 0.15	1.82 ± 0.11
%BOP (%)	20.73 ± 15.38	13.72 ± 10.11
PISA (mm^2^)	235.47 ± 184.49	148.34 ± 109.48
PCR (%)	54.88 ± 26.85	48.00 ± 25.22
Vitality in the morning	0.35 ± 0.13	0.37± 0.12
Vitality in the afternoon	0.39 ± 0.14	0.34 ± 0.13
Vitality in the evening	0.39 ± 0.15	0.40 ± 0.15
Mental activity	0.45 ± 0.18	0.44 ± 0.13
PHQ-9 score	3.03 ± 3.21	3.03 ± 3.64
BDI score	5.93 ± 5.39	4.85 ± 6.49

Values are presented as mean ± SD.

**Table 2:** Correlation between score of self-report psychological questionnaire and vitality or mental activity.

		Vitality in the Morning	Vitality in the Afternoon	Vitality in the Evening	Mental Activity
PHQ-9 score	ρ	−0.221	−0.182	−0.107	−0.128
	*p* value	0.025	0.055	0.185	0.215
BDI score	ρ	−0.219	−0.098	0.016	−0.169
	*p* value	0.025	0.196	0.448	0.149

Spearman’s rank correlation coefficient.

**Table 3:** Correlation between mental activity, changes in vitality in the morning, PHQ-9, and BDI score and oral health status.

		Change in Mean PPD	Change in %BOP	Change in PISA	Change in PCR
Mental activity	ρ	−0.265	−0.037	0.003	−0.210
	*p* value	0.049	0.410	0.492	0.096
Change in vitality in the morning	ρ	0.148	−0.374	−0.300	0.207
	*p* value	0.182	0.009	0.030	0.099
Change in PHQ-9 score	ρ	0.008	−0.054	−0.053	0.084
	*p* value	0.480	0.370	0.374	0.303
Change in BDI score	ρ	0.010	0.055	0.050	0.152
	*p* value	0.476	0.369	0.381	0.174

Spearman’s rank correlation coefficient.

## Data Availability

No new data were created or analyzed in this study. Data sharing is not applicable to this article.
